# Detection of an Antagonist Bound to the Neurokinin a Receptor in Styrene–Maleic Acid Lipid Particles by ^19^F Ultrafast Magic‐Angle Spinning Nuclear Magnetic Resonance Spectroscopy

**DOI:** 10.1002/cbic.202500963

**Published:** 2026-03-23

**Authors:** Samuel Seidl, Aditya Prasad Patra, Chengkang Li, Johanna Becker‐Baldus, Stefanie Kaiser, Christoph Reinhart, Clemens Glaubitz

**Affiliations:** ^1^ Institute for Biophysical Chemistry and Center for Biomolecular Magnetic Resonance (BMRZ) Goethe University Frankfurt Frankfurt am Main Germany; ^2^ Max Planck Institute of Biophysics Frankfurt am Main Germany; ^3^ Institute for Pharmaceutical Chemistry Goethe University Frankfurt Frankfurt am Main Germany; ^4^ UMass Chan Medical School University of Massachussetts Worcester Massachusetts USA; ^5^ Children's Hospital Zurich and Children's Research Center Pediatric Cancer Metabolism Laboratory and Division of Division of Oncology University of Zurich Zurich Switzerland; ^6^ BioCopy AG Basel Switzerland

**Keywords:** ^19^F, GPCR, neurokinin A receptor, SMALP, ultra‐fast MAS NMR

## Abstract

G protein‐coupled receptors (GPCRs) are major pharmacological targets, with ≈34% of FDA‐approved drugs acting on this protein family. The frequent incorporation of fluorine into small‐molecule ligands enables the use of ^19^F nuclear magnetic resonance (NMR) as a sensitive and site‐specific method to probe ligand behaviour. In this case study, we investigate the fluorinated antagonist GR159897 bound to the membrane‐embedded neurokinin A receptor reconstituted in detergent‐free styrene–maleic acid lipid particles (SMALPs) using ^19^F ultrafast magic‐angle spinning (MAS) solid‐state NMR. We demonstrate that the environmental sensitivity of ^19^F NMR resolves distinct ligand populations within the SMALP system and enables detection of receptor‐associated ligand, highlighting the utility of ^19^F solid‐state NMR for probing GPCR–ligand interactions.

## Graphical abstract

The detection of a fluorinated antagonist bound to the neurokinin A receptor by ^19^F ultrafast MAS NMR is demonstrated. Therefore, the receptor is purified by detergent‐free extraction by styrene maleic acid copolymer encapsulation and subsequently characterised.

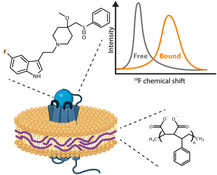

## 1 Introduction

Drugs targeting G protein‐coupled receptors (GPCR) account for ≈34% of all FDA‐approved pharmaceuticals, underscoring the continued demand for new compounds [1, 2]. Drug discovery in this field has been significantly accelerated by high‐resolution structural information from X‐ray crystallography and single‐particle cryo‐electron microscopy, which provide the spatial framework required for modern computational approaches [3, 4]. In parallel, nuclear magnetic resonance (NMR) spectroscopy has delivered essential insights into GPCR activation mechanisms and conformational dynamics [5, 6, 7, 8, 9, 10, 11, 12].

^19^F NMR has become particularly popular because the ^19^F nucleus is highly sensitive to changes in its local chemical environment, making it an excellent probe for monitoring receptor activation, ligand binding, and ligand binding modes and dynamics. Consequently, protein‐observed solution‐state NMR has been used to resolve the conformational ensembles of GPCRs at different stages of activation and to reveal lipid‐dependent effects on GPCR activation [6, 7, 8, 10, 13, 14]. These insights rely on the detection of a fluorine label chemically conjugated to the protein. While this approach benefits from the high sensitivity of ^19^F and the absence of background signals, leading to relatively simple spectra, the fluorine probe must always be introduced artificially at strategically selected positions.

Ligand‐observed ^19^F NMR has enabled the characterization of ligand dynamics within the binding pocket in solution [15]. However, ligand‐observed ^19^F NMR is more commonly employed in drug screening, where the free ligand population is typically monitored [16, 17, 18, 19]. Binding events or competition with other ligands are inferred from changes in the intensity of the free‐ligand signal. This spectroscopic strategy has proven highly effective in fragment‐based drug discovery, helping to narrow the chemical space of potential ligand structures and identify promising lead compounds [20, 21].

Most NMR studies on GPCRs have been performed in solution using membrane mimics such as detergent micelles or nanodiscs [13, 22]. However, because of their size and intrinsic conformational dynamics, GPCRs remain challenging targets for NMR. Fewer studies have employed solid‐state NMR, in which magic‐angle spinning (MAS) averages size‐dependent anisotropic interactions, reducing sensitivity to molecular size and to the specific type of membrane mimic used [23, 24, 25]. More recently, protein‐observed ^19^F MAS NMR has been applied to investigate how membrane architecture influences the conformational ensembles of GPCRs [26]. In addition, a limited number of studies have used isotope‐labelled ligands as probes to obtain more detailed insight into GPCR‐ligand binding modes [27, 28, 29, 30].

Here, we investigate the feasibility of ^19^F ultrafast MAS NMR for detecting a fluorinated high‐affinity ligand bound to a GPCR. Ultrafast MAS, during which samples are spun at 100 kHz or higher, is a recent technological advancement in solid‐state NMR originally introduced to enable efficient proton detection in solids and large biomolecular systems [31, 32]. More recently, it has been demonstrated that ^19^F also benefits from faster spinning rates due to more effective ^1^H decoupling under these conditions. The development of new approaches for characterizing ligand–protein interactions involving fluorinated compounds is therefore highly desirable. Drug screening studies make regular use of fluorinated compounds as a direct probe or as a competitor. More importantly, the number of fluorinated drugs being approved by the FDA is increasing [33]. Fluorinated compounds exhibit favourable pharmacokinetic properties, e.g., increased bioavailability or oxidative stability [34]. Consequently, the approach presented here is expected to complement existing methods for the characterization of current and emerging drugs and their protein–ligand complexes.

**FIGURE 1:**
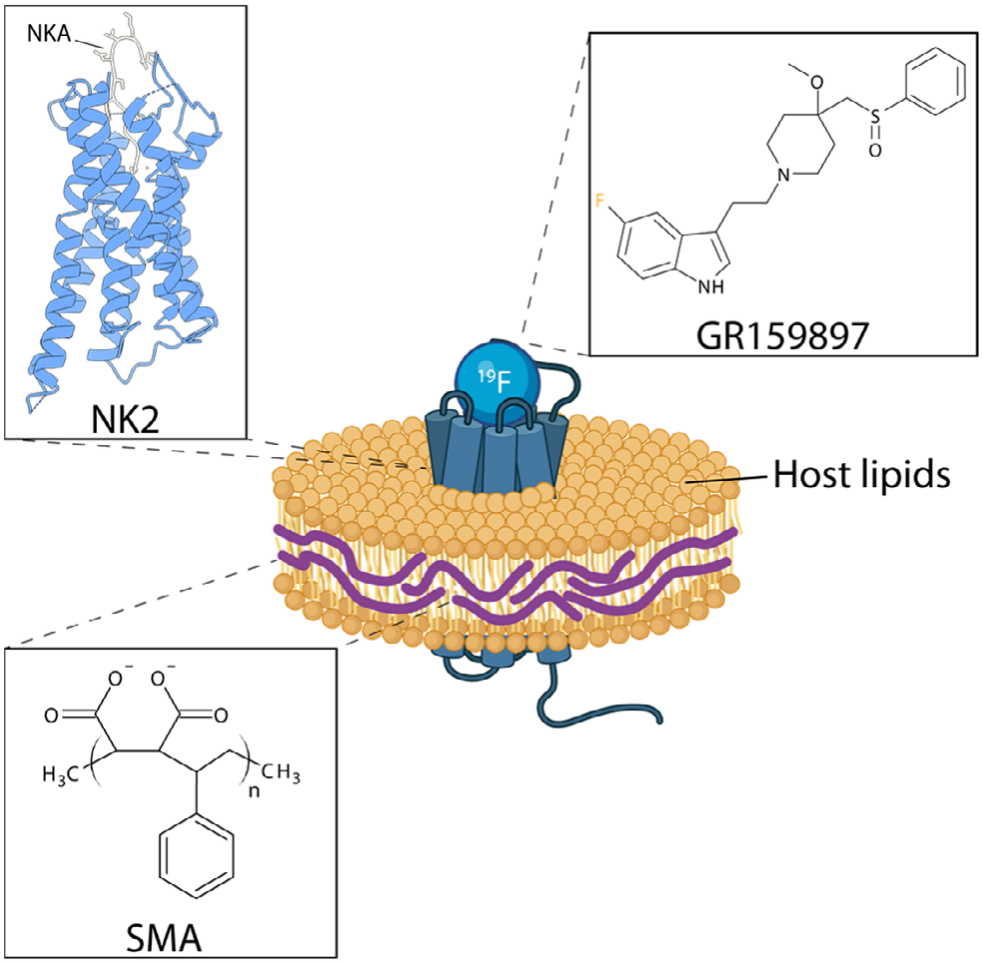
The neurokinin A receptor in a SMALP (schematic). The neurokinin A receptor (in blue in complex with neurokinin A (NKA; PDB ID 7XWO) reconstituted with the help of styrene maleic acid (SMA) copolymer and bound to GR159897. This figure was partially created with BioRender [35].

As a model system, we selected the rat neurokinin‐2 (NK2) receptor from the tachykinin receptor family together with its antagonist GR159897 (Figure 1). The NK2 receptor (also called NK2R or TACR2), whose structure was recently solved [36], is activated by its main endogenous agonist neurokinin A. It primarily mediates smooth muscle contraction, nociception, and inflammatory and autonomic regulation [37]. NK2 signals through G_q/11_‐mediated pathways. Similar to its successfully drugged relative, the substance P receptor (NK1), NK2 displays promiscuous expression across diverse cell types and tissues [37, 38]. The pathophysiological relevance of NK2 is underscored by the clinical development of several of its ligands. Ibodutant, for instance, advanced to phase 3 clinical trials for the treatment of irritable bowel syndrome, highlighting the role of NK2 in gastrointestinal inflammatory pathways [39]. Likewise, saredutant was evaluated clinically for depressive disorder, demonstrating NK2's involvement in central nervous system function [40]. The fluorinated NK2 antagonist used in this study, GR159897 (Figure 1), has been shown to inhibit NK2‐mediated bronchoconstriction in animal models [41, 42]. These examples illustrate the broad functional spectrum of NK2, which varies substantially with its site of expression. Recent interest in NK2 has been reignited by a study demonstrating that, in contrast to the previously described antagonistic effects, NK2 agonism increases metabolic expenditure and suppresses appetite. This work also involved the development of novel NK2 agonists, paving the way for new therapeutic strategies targeting obesity and type 2 diabetes [43].

NK2 was encapsulated into styrene maleic acid copolymer lipid particles (SMALP) (Figure 1) [44, 45, 46]. SMALPs provide a major advantage in that membrane proteins can be extracted and stabilized without the use of detergents, which can destabilise membrane proteins. During solubilisation, the SMA copolymer inserts into the native membrane, excising nanoscale lipid patches that are subsequently encapsulated to form soluble, discoidal particles. These particles preserve the protein's native lipid environment and can be readily purified. Unmodified SMA is widely available and commonly used [47]. Since the introduction of SMA, additional polymers—such as diisobutylene maleic acid (DIBMA) and various modified SMA derivatives—have been developed, reflecting the broad interest in detergent‐free membrane protein extraction [48, 49, 50, 51]. Moreover, SMA or polymer purifications show different protein‐dependent characteristics in terms of size and protein activity [48, 52, 53, 54, 55, 56, 57, 58, 59]. Initially, it was assumed that SMA only form discoidal particles. However, a recent study has shown that SMALPs can adopt a variety of morphologies [60]. This highlights the need to further expand the knowledge base surrounding protein‐SMALP systems. So far, the number of GPCR‐SMALP preparations are limited with reports including, i.e., the adenosine A_2a_ receptor, β‐adrenergic receptor and dopamine receptors [61, 62, 63, 64, 65]. In combination with solid‐state NMR, SMA, and DIBMA have been used [66, 67].

**FIGURE 2:**
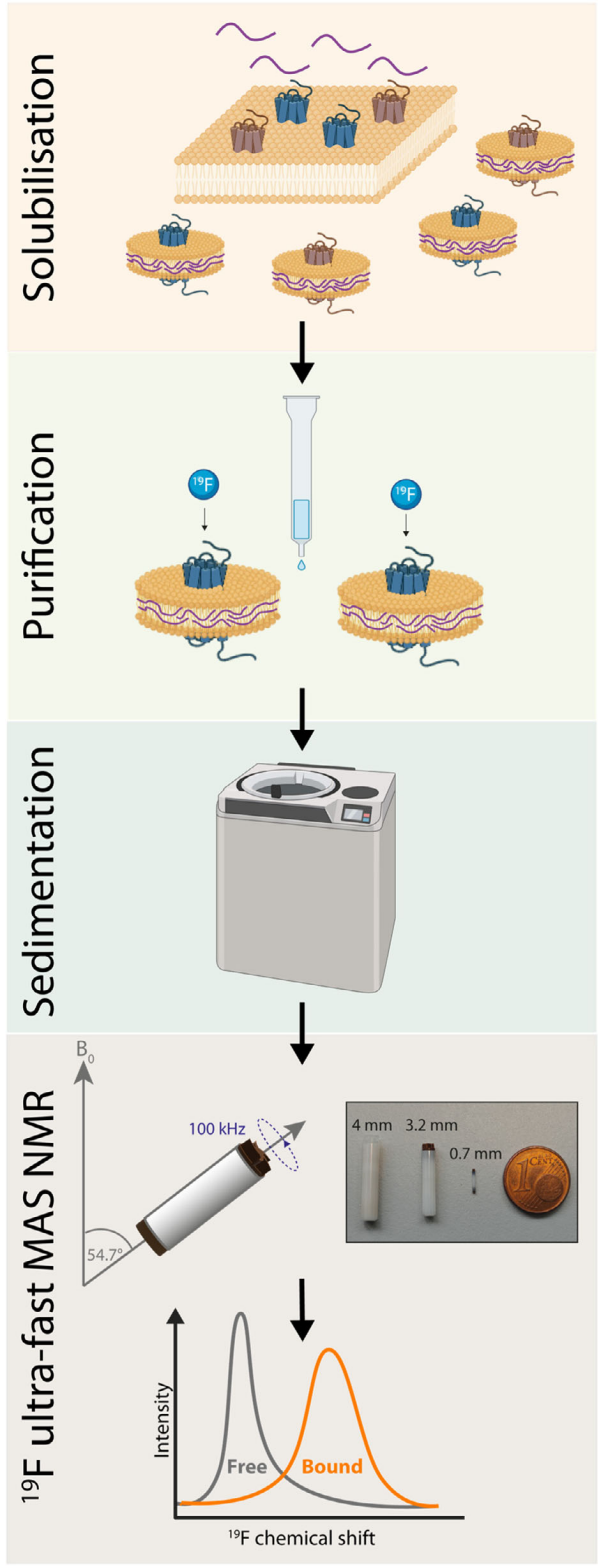
Pipeline for the characterization of GPCR‐ligand complexes by ligand‐observed ^19^F ultrafast MAS NMR. The GPCR is expressed in *P. pastoris*, blue: NK2, brown: other membrane proteins. After expression, the prepared membranes are solubilized by SMA addition. The NK2 containing SMALPs are purified. After purification, the ligand is added and sedimented into the MAS rotor and ^19^F NMR measurements are performed under ultrafast MAS. This figure was partially created using BioRender [68].

Here, we demonstrate a detergent‐free purification of NK2 from yeast, its encapsulation in SMALPs containing native host lipids, and the use of these particles for ^19^F ultrafast MAS NMR spectroscopy (Figure 2). The resulting NMR spectra show that it is possible to detect the bound fluorinated antagonist GR159897. Moreover, the high environmental sensitivity of ^19^F enabled us to distinguish clear differences between the SMALP‐associated and receptor‐bound populations.

## 2 Results and Discussion

### 2.1 Purification of NK2‐SMALP

**FIGURE 3:**
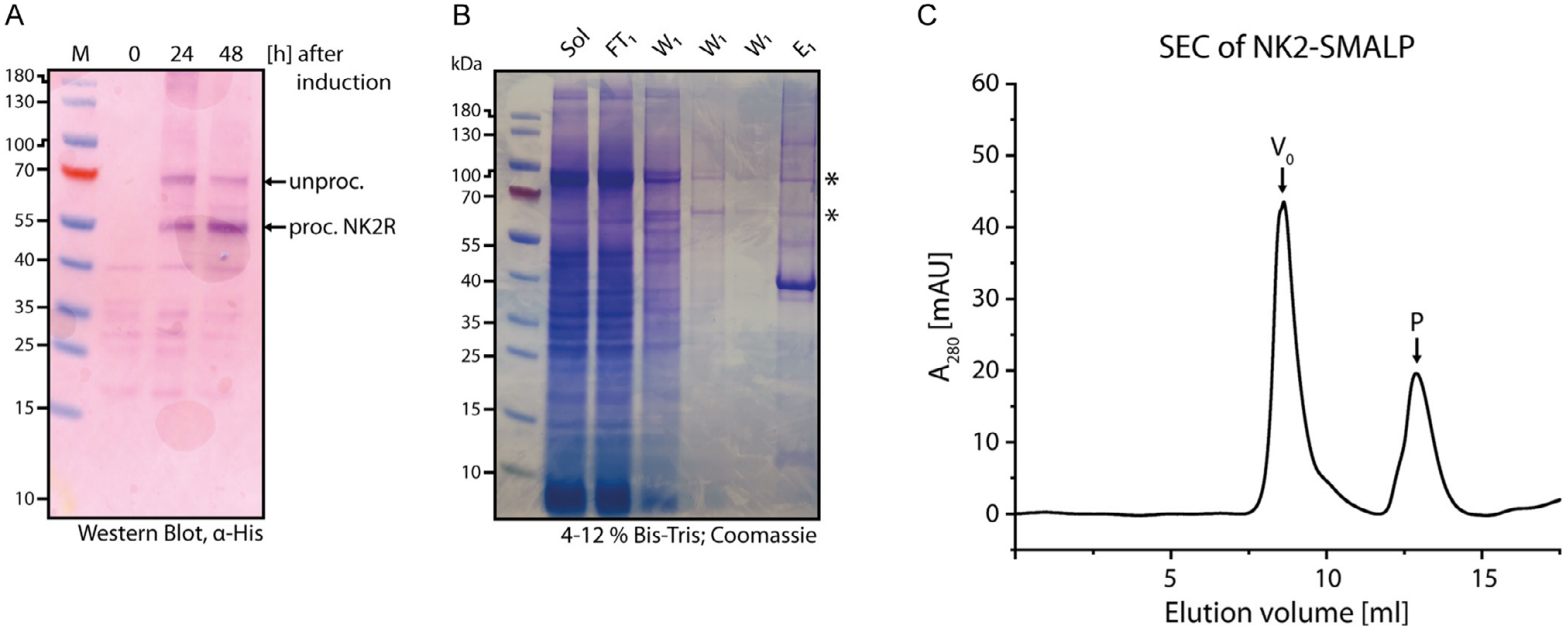
Expression and purification of NK2‐SMALP. (A) Western Blot of NK2 expression. During expression, culture samples are taken before induction, 24 h and 48 h after induction. Membranes are prepared and a Western blot is performed with detection of the His‐tag. (B) SDS‐PAGE of NK2‐SMALP purification. Applied on the gel are the solubilisation (Sol), the flow‐through of the IMAC purification (FT), different fractions of Wash 1 (W_1_) and the eluate (E_1_). Asterisks mark the NK2 bands observed in the Western blot. (C) Size‐exclusion chromatogram of NK2‐SMALP. ≈500 µg of protein was applied on the column. *V*_0_ indicates the void volume. Peak P was used for LC‐MS analysis.

Expression and purification of NK2 in insect cells has been described previously [36]. Here, we report its expression in yeast and detergent‐free encapsulation in lipid‐containing SMALPs. Rat NK2 was expressed in *Pichia pastoris*, which can be beneficial for the expression of eukaryotic membrane proteins as it has an endomembrane system with a more native folding machinery and more native‐like post‐translational glycosylation of the protein, compared to *E. coli*. The gene of interest was stably integrated into the genome. After selection of a clone with high copy number and thus high expression levels, the clone was kept and freshly streaked for expression. For expression analysis by Western blot, 10 mL of the culture were taken after one or 2 days. Small‐scale lysis by glass beads was performed and the membranes were isolated by ultracentrifugation. Thereafter, the membranes were resuspended in buffer and 30 µg of total membrane protein was used for gel electrophoresis. Western blot analysis revealed two bands at 55 kDa and 67 kDa 24 h after methanol induction (Figure 3A). These bands correspond to the expected molecular weights of the receptor without (55 kDa) and with signal peptide (67 kDa) , respectively. After 48 h, the relative intensities shifted in favour of the processed receptor, while the total receptor level in the membrane fraction remained unchanged.

For purification the cells were subjected to high‐pressure homogenization. Then, the membrane was isolated by ultracentrifugation and solubilised using SMA followed by immobilised metal ion affinity chromatography. The resulting NK2‐SMALPs were first subjected to SDS‐PAGE (Figure 3B). The obtained bands deviated slightly from those observed in the Western blot. The Coomassie stained gel shows an intense band at 40 kDa next to the protein size matching bands described for the Western Blot. The eluate was subsequently subjected to size‐exclusion chromatography (Figure 3C). The resulting chromatogram displays a large void volume peak (*V*_0_) and a peak (P) at 12.86 mL, corresponding to a particle with an estimated discoidal diameter of ˜ 10 nm, consistent with the size of other discoidal assemblies such as nanodiscs [56, 69]. SDS‐PAGE of this peak shows one species appearing at 40 kDa (Figure S1).

Due to its unexpected migration behaviour on SDS‐PAGE, the species P isolated by size exclusion chromatography (SEC) was subjected to tryptic digestion followed by liquid chromatography mass spectrometry (LC‐MS) analysis. The sequence coverage obtained was 15.81%. The recovered peptides included the tobacco virus etch protease (TEV) cleavage site at the N‐terminus as well as C‐terminal fragments of the receptor and the fused biotinylation domain (see Supporting Information for further details) confirming that this population contains the NK2 receptor. Since our targeted LC‐MS analysis only confirmed the presence of the receptor within P, the additional presence of a foreign protein cannot be ruled out.

For gel electrophoresis, the sample was solubilized with SDS, which promotes protein unfolding. To our knowledge, it remains unclear whether the SMA polymer remains associated with the protein and how this association depends on SMALP morphology. Membrane proteins typically display increased mobility in SDS‐PAGE due to enhanced SDS binding, which leads to a higher net negative charge and consequently faster migration. Based on this behaviour, we speculate that NK2 may migrate even further down the gel if the strongly negatively charged SMA polymer—owing to its maleic acid moieties—remains attached to the protein. A similar downward shift has been observed for other proteins expressed in *P. pastoris*; however, this phenomenon was not discussed further [56, 65]. We conclude that the corresponding SEC peak represents intact discoidal SMALPs that appear to be stable in SDS. In total, only 50 µg out of 500 µg of protein were recovered after SEC.

So far, NK2 was expressed in insect cells and purified using detergent for structure determination [36]. Besides, SMALP preparations have been shown for other well characterised GPCRs [61]. Here, we have now a detergent‐free preparation of rat NK2 receptor in SMALPs filled with host lipids (main components are phosphatidylserine, phosphatidylcholine, and phosphatidylethanolamine) [70].

### 2.2 Characterization of NK2‐SMALP

**FIGURE 4:**
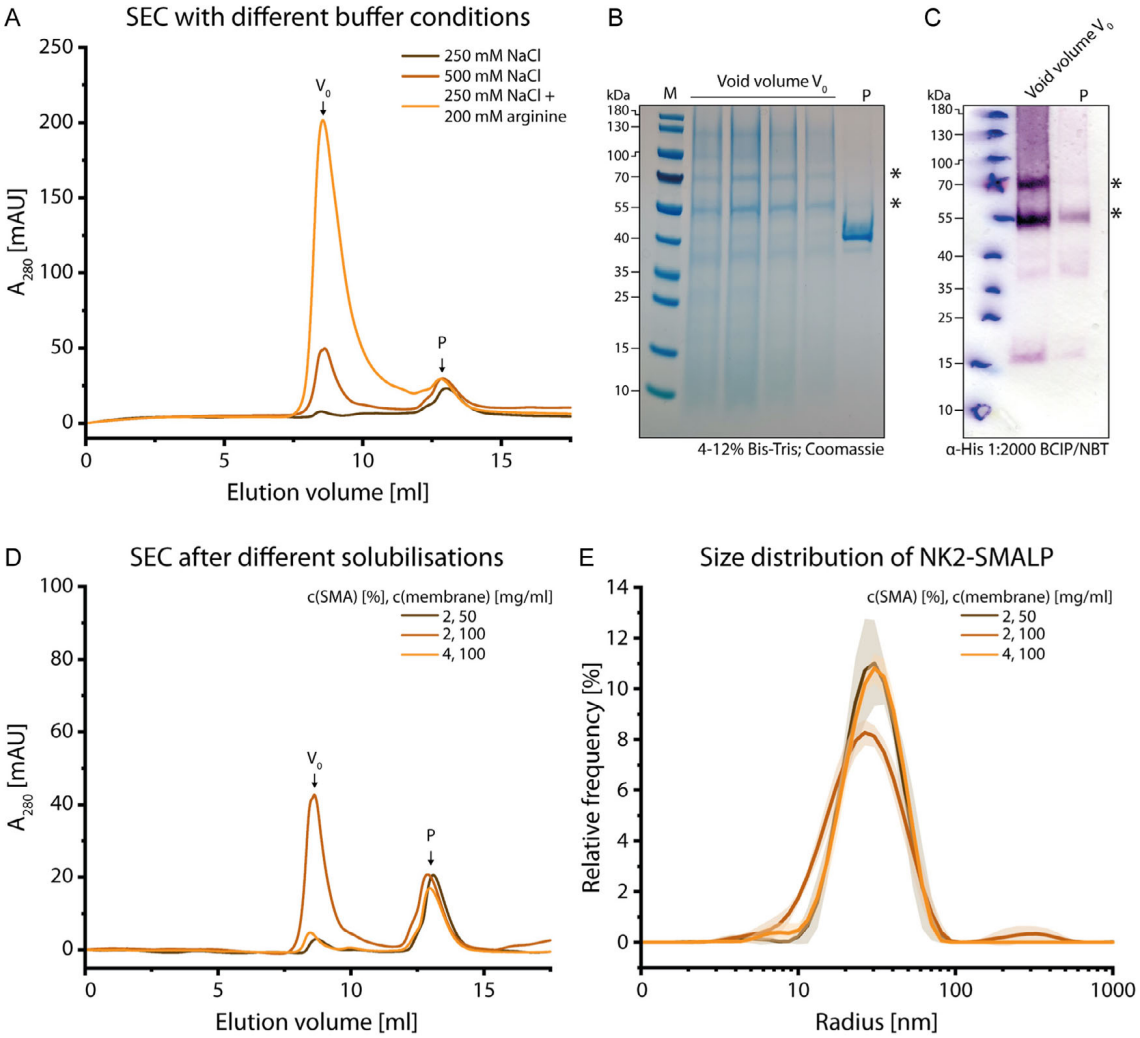
Characterization of NK2‐SMALP. (A) SEC of NK2‐SMALP using different salt conditions or buffer additives. The SEC column was equilibrated with lower NaCl concentration (brown), standard salt conditions (light brown) or low NaCl concentration and additional 200 mM arginine (orange) prior to the application of 500 µg protein. The peaks from the SEC run with arginine were collected and analysed *via* SDS‐PAGE (B) and Western blot (C). For SDS‐PAGE, the peak fraction was concentrated due to the low sample amount. Asterisks mark NK2 bands as seen in Figure 1A. (D) SEC of NK2‐SMALP solubilised with 2% SMA with a low membrane concentration (brown), standard solubilisation conditions (light brown) or with doubled SMA concentration (orange). (E) DLS of differently solubilised NK2‐SMALP with the conditions mentioned before. Size measurements were performed with a rebuffered sample (light brown) or directly after IMAC (brown and orange).

The generated NK2–SMALPs were further characterized for size and homogeneity using analytical SEC and dynamic light scattering (DLS) (Figure 4). To address the poor sample recovery after SEC as described above, we assessed whether nonspecific interactions were responsible for particle retention on the column. Therefore, we varied the SEC buffer conditions (Figure 4A).

Lowering the salt concentration decreased the population *V*_0_ eluting in the void volume, but the peak P at 12.86 mL remained unchanged in both retention volume and intensity. This suggests that overall sample recovery decreases. Previous studies have reported improved SEC recovery of SMALPs and other lipid‐based nanoparticles when arginine is included in the buffer, indicating that these additives help to reduce nonspecific interactions [71, 72, 73]. Here, addition of arginine led to a stark increase of the void volume peak *V*_0_ recovering nearly everything of the applied sample (Figure 4A). Analysis of the peak fractions from this condition showed that the peak P at 12.86 mL consisted mainly of the protein band at 40 kDa after IMAC (Figure 4B). This band was absent in the void volume fraction. In contrast, the void volume fractions from the IMAC eluate contained bands at 55 kDa and 70 kDa and a smear below 40 kDa. In order to specifically detect NK2, Western blot against the His‐Tag was performed on the SEC fractions. Here, the void volume peak *V*_0_ showed two intense bands at 55 kDa and 70 kDa within a smear between 180 and 55 kDa. This pattern was identical to the Western blot of the expression analysis (Figure 3A). The peak P at 12.86 mL had fainter bands at the same positions.

The existence of two different SEC populations, which show the same bands on Western blots but different SDS‐PAGE migration behaviour point to SMA particles which do not only differ in size but also in their structural properties. Western blot of the void volume fraction *V*_0_ yielded bands which match exactly those observed for the protein in nonpurified yeast membranes (Figure 3A). This means that these SMALPs from the void volume *V*_0_ behave differently to those obtained from the population obtained for P at 12.86 mL during SDS‐PAGE as they seem to dissolve in SDS. Recent advances have shown that SMALPs are not only discoidal but can also exist in different architectures such as ‘polymer‐remodelled liposomes’, ‘polymer−lipid mixed micelles’, or ‘lipid‐doped polymer micellar aggregates’ [60] corroborating our observations.

As SEC analysis exposed two SMALP species, which most likely differ in their morphology, solubilisation conditions were altered to potentially homogenize them. It has been reported, that increasing the polymer‐to‐lipid ratio decreases the size of the SMALP when titrated to synthetic liposomes [55, 60]. Therefore, the polymer‐to‐membrane ratio was varied by either doubling the concentration of SMA used or by decreasing the membrane concentration. The latter condition was used in order to illuminate potential influences of varying membrane concentration on the solubilisation behaviour and to monitor variations during IMAC. SEC was employed to determine whether increasing the amount of SMA leads to smaller particle sizes and, consequently, a higher yield of particles separable by SEC (Figure 4D). Overall, virtually no shift in the chromatographic peaks was observed, leaving the chromatogram essentially unchanged. However, the void‐volume peak (*V*_0_) was reduced, while the intensity of peak P at 12.68 mL remained unchanged under both conditions. This suggests that a larger fraction of particles interacts with the column. Therefore, for *P. pastoris* membranes, no increase in the amount of potential discoidal particles was observed by SEC. For the conditions tested, no size reduction has been observed.

Since neither buffer nor solubilisation conditions improve SEC separation, preparative SEC was omitted for further analysis. Consequently, the final sample contains populations *V*_0_ and P, with *V*_0_ being the dominant form. Therefore, although the presence of a foreign protein in the P population cannot be excluded, it would likely contribute only minimally to the overall sample. In order to gain insight into the SMALP size distribution as a function of solubilisation conditions, DLS measurements were performed (Figure 4E). Under initial solubilisation conditions (100 mg/ml membrane solubilized with 2% SMA), one main population with a particle size of 25.2 ± 0.8 nm emerged during the measurements, which would be in agreement with a SEC peak eluting in the void volume. In a previous study, particles with a 10 nm size were extracted, when a protein was solubilised from *P. pastoris* membranes [56]. Consequently, it can be stated that the particle size can vary significantly and might be protein dependent. The observed size distribution is characterized by a polydispersity index of 0.35 ± 0.06, indicating acceptable homogeneity.

Contrary to previous studies [55, 60], increasing the total amount of SMA increased particle size by ≈4 nm, while the polydispersity index decreased towards monodispersity (Table S1). Currently, there is no model or study to explain this behaviour when proteins are solubilized from their host membranes. An explanation could be that another parameter than the polymer amount controls the shift from other SMA architectures to discoidal particles. For example, in case of alternative SMALP architectures such as ‘polymer‐remodelled liposomes’ [60], an increase of SMA could potentially lead to larger particles with increased monodispersity. Consequently, this optimisation step can be considered for structural studies, e.g., cryo‐electron microscopy. However, this needs to be further elucidated due to the polydisperse nature of the polymer itself.

Since an increased SMA concentration did not lead to the possibility of preparative SEC, initial solubilisation conditions were kept in order to keep SMA consumption low. More importantly, it is likely that the majority of the intact protein is within the same SMA entity based on size distribution, polydispersity index and SEC retention behaviour.

### 2.3 Activity and Stability of NK2‐SMALP

**FIGURE 5:**
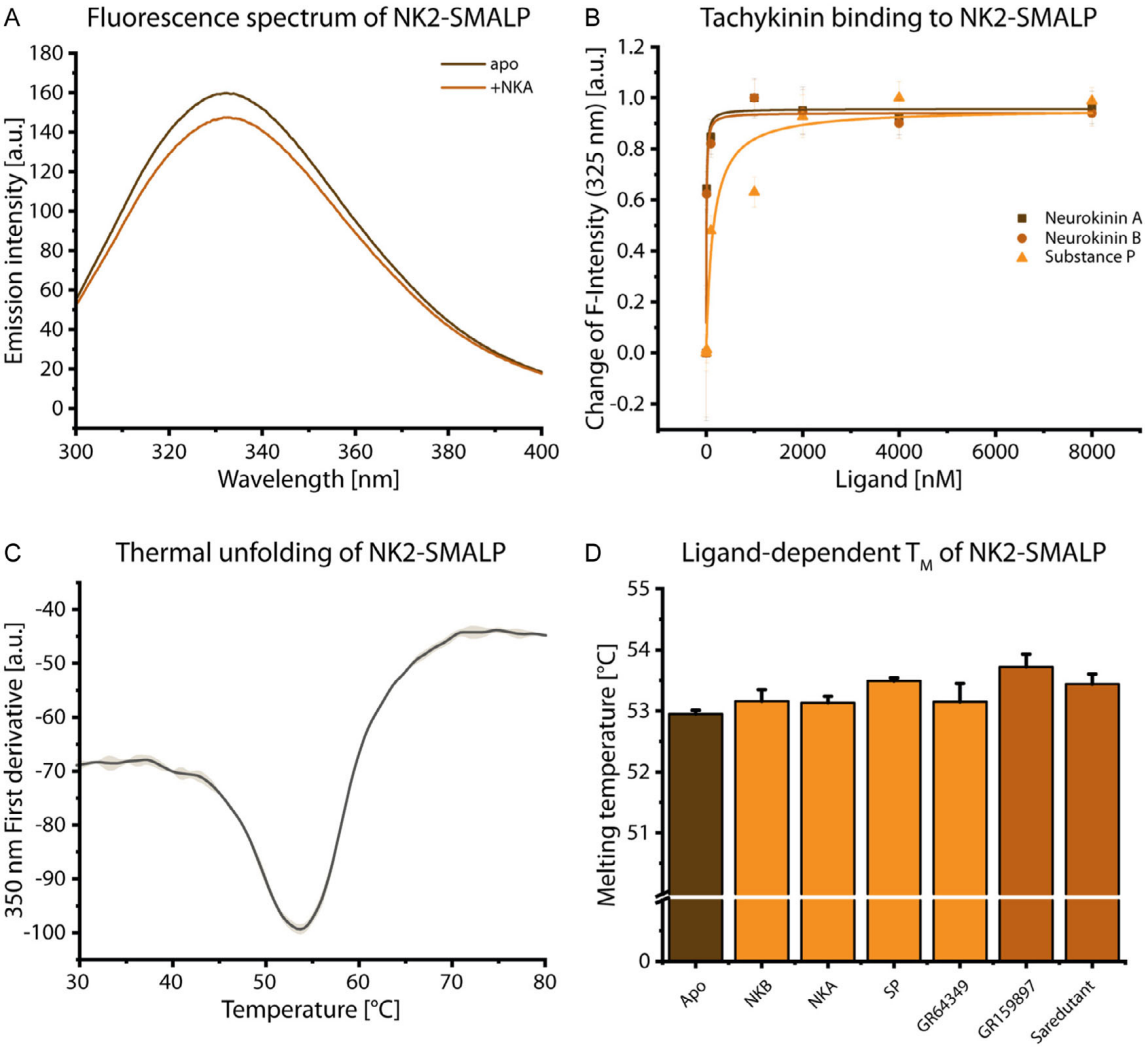
Activity of NK2 in SMALP. (A) Tryptophan fluorescence of NK2‐SMALP. An emission spectrum (excitation at 280 nm) of the tryptophans in NK2 was recorded without (brown) and with the agonist neurokinin A (light brown). (B) Ligand‐dependent tryptophan fluorescence of NK2. The different endogenous ligands neurokinin A (brown points), neurokinin B (light brown) and substance P (orange) were titrated to the protein. Tryptophan fluorescence at 325 nm was measured and plotted against the titrated ligand concentration. (C) Thermal stability of NK2 in SMALP measured by nanoDSF. The derivative of the measured tryptophan fluorescence at 350 nm was monitored in dependence of the temperature. (D) Ligand‐dependent melting temperatures of NK2‐SMALP. NanoDSF measurements were performed in slight excess of agonists (orange) or antagonists (brown) and compared to the apo protein (brown). Error bars show the deviation of measurement triplicates from *n* = 1.

To further characterise NK2 within the SMALP, activity and stability of the protein were probed (Figure 5). Here, activity of NK2 was confirmed by ligand binding. Therefore, changes in tryptophan fluorescence of the protein were monitored to circumvent unspecific signals of other homogenous, ligand‐observed binding assays (Figure 5A,B). This approach proved to be successful for the adenosine A_2_a receptor in SMALPs [74]. For NK2, 10 tryptophans contribute to the signal.

The fluorescence spectrum of the apo receptor exhibits a broad emission spectrum ranging from at least 300 nm to 380 nm (Figure 5A), which is comparable to beforementioned reports of other GPCRs [74]. Addition of the endogenous agonist NKA in a slight stoichiometric excess led to a reduction of fluorescence. Based on this reactivity, the endogenous ligands NKA, NKB, and SP were titrated and the change was monitored (Figure 5B). GR159897 was excluded in this titration due to the fluorescence of its fluoroindole moiety. The fluorescence intensity decreased strongly at lower concentrations of ligand and changes were minimal above 4 µM indicating saturation and doubling the ligand concentration did not lead to larger changes. Subsequently, the data were fitted using a one‐site binding model to determine apparent *K*_D_ values (Table S2). The *K*_D_ values for NKA and NKB were similarly low, at ≈7 nM, whereas the affinity for SP was at least an order of magnitude higher.

Consequently, it was possible to obtain affinity values which are consistent with the reported binding preferences of NK2 to NKA, NKB, and SP and in the same order of magnitude [75] demonstrating an active NK2‐SMALP preparation.

In contrast to the study on the adenosine A_2A_ receptor SMALPs, NK2 fluorescence did react to all three endogenous agonists, whereas A_2A_ receptor fluorescence was only modulated by an antagonist. Consequently, the feasibility of this assay can vary from receptor to receptor. Several limitations can be observed from the spectra: On the one hand, the changes of the raw signal were small. Thus, the obtained *K*_D_ values show stronger deviations. Moreover, due to the high affinity of the endogenous ligands, the used receptor concentration was chosen as small as possible. Still, the receptor concentration was much higher than the anticipated affinity for NKA and NKB posing a suboptimal assay condition. For SP, the receptor concentration was below the expected *K*_D_ leading to a more typical binding curve. Therefore, the affinities obtained need to be considered with care and cannot report quantitatively about the binding. However, it can be successfully demonstrated, that the receptor is active with affinities in the nanomolar range and that this fluorescence assay is a low‐threshold approach to examine the binding competency of GPCRs for subsequent studies.

To combine further characterization of binding with the characterization of the protein, thermal shift assays can be applied. NanoDSF measurement of NK2 in SMALPs reveals a cooperative unfolding at 54.36 ± 1.82°C (Figure 5C). Most likely, the lipid environment stabilises the protein which was also observed for other proteins [26, 76, 77].

For some GPCRs, ligand binding led to an increase of protein stability [26, 77]. Yet, the obtained melting temperature is ligand‐dependent. Here, a series of agonists (endogenous and artificial) as well as antagonists was added with a 1.25× stoichiometric excess before the unfolding was monitored (Figure 5D). For all ligands used, a slight increase in melting temperature was observed. The melting temperatures were different for each ligand (Table S3). However, only for SP and GR159897 the increase of ≈0.5 K was greater than the variance compared to the apo receptor.

In contrast, other reports state stabilisations of GPCRs of up to 10 K [77, 78]. However, these measurements were made in detergent. There is a possibility that a membrane environment dampens the additional, potentially stabilising effect of ligand binding [76].

### 2.4 Detecting GR159897 Bound to NK2‐SMALPs by ^19^F Ultrafast MAS

**FIGURE 6:**
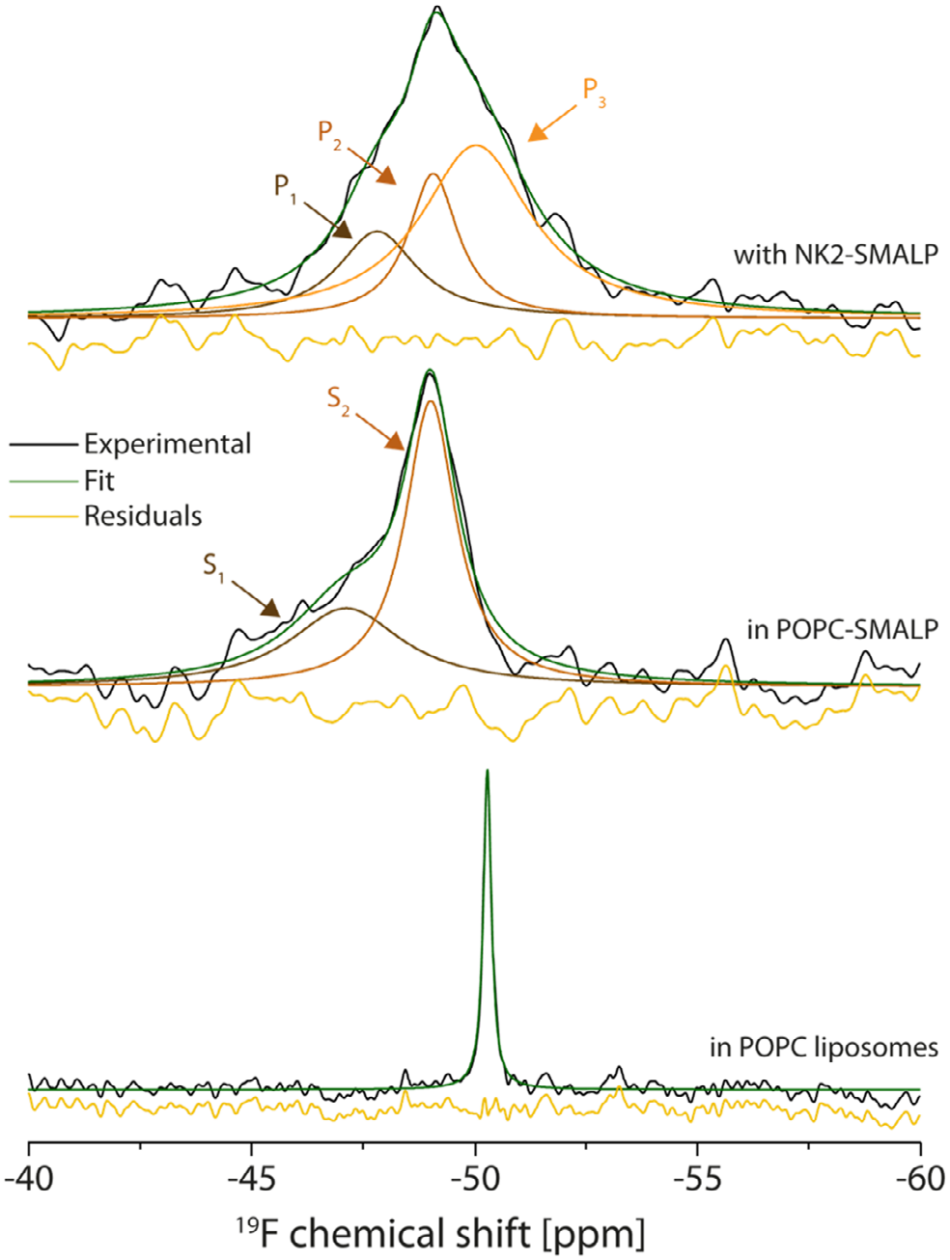
GR159897 binding to NK2 in SMALP. 1D ^19^F NMR of GR159897 in POPC liposomes (bottom spectrum), in POPC‐SMALP (middle spectrum) and bound to NK2 in SMALP (top spectrum). The measurements were performed at ≈25°C and at 100 kHz MAS at 800 MHz ^19^F Larmor frequency. The complex lineshape of the ligand in SMALP and bound to the protein was deconvoluted and the deconvoluted spectrum was overlayed (green spectra). The assignment of the labelled resonances is explained in the discussion and summarised in (Table S5).

The size characterization revealed SMALPs with a size of 25 nm, which are larger than previously published SMA solubilisations in *Pichia pastoris* [56]. The resulting large NK2‐SMALPs are therefore a suitable target for ssNMR spectroscopy after sedimentation. ^19^F‐MAS NMR allows direct and background‐free detection of GR159897. Moreover, ultrafast sample rotation (>100 kHz) improves resolution and sensitivity. The very small sample volume of the used 0.7 mm MAS rotors accommodates the typically small sample amounts available for GPCR preparations. As the SMALP platform is yet unexplored for ligand‐observed NMR, a comparison and stepwise characterization of the ligand is performed starting from POPC liposomes, to the ligand in POPC‐SMALPs and finally the ligand bound to the receptor in SMALPs (Figure 6).

Liposomes containing 300 µM GR159897 (corresponding to a 1:15 ligand‐to‐lipid molar ratio) exhibit a single narrow peak at −50.3 ppm (Figure 6, bottom). The absence of a complex lineshape indicates a single predominant population of the ligand. Due to its hydrophobicity (log*P* = 2.37), the ligand should be within the lipid bilayer. When the same amount of ligand is added to POPC‐SMALPs, which are then sedimented into the rotor, a down‐field shifted and a more complex lineshape is observed (Figure 6, middle). A Lorentzian deconvolution was applied in order to fit the signal with the least number of peaks. Two Lorentzian lines, S_1_ and S_2_, were sufficient to match the spectrum. S_1_ occurs at −47.08 ± 0.07 ppm and S_2_ at −49.02 ± 0.01 ppm, with S_1_ being broader with lower integral intensity (Table S3). Compared to the ligand in liposomes, the much broader peaks in the POPC‐SMALP sample indicate a larger ligand heterogeneity, slower dynamics or both. Lipid order and dynamics are altered in nanodiscs or SMALPs [79, 80], which can affect the ligand distribution within such preparations. The overall downfield shift of both peaks relative to the POPC spectrum is likely caused by the increased polarity introduced by the maleic acid subunits of the polymer. Compared to S_1_, signal S_2_ appears at higher field and with greater intensity. The high‐field shift reflects the hydrophobic lipid environment, which can also accommodate more ligands. Signal S_2_ is therefore assigned to the lipid‐associated ligand population.

In contrast, the more downfield‐shifted signal S_1_ likely corresponds to ligands that are associated with, or positioned closer to, the SMA polymer rather than embedded within the lipids. The smaller population size further indicates that the ligand preferentially partitions into the lipid phase. This secondary population is most likely a consequence of the excess ligand added to allow direct comparison with the liposome preparation and to ensure adequate signal‐to‐noise during measurement.

When NK2‐SMALPs were incubated with an ≈ 1.1‐fold molar excess of GR159897 (≈80 µM), the resulting complex lineshape broadened noticeably (Figure 6, top). Deconvolution of the spectrum required three peaks (P_1_–P_3_).

The most downfield‐shifted peak (P_1_) differs markedly from the corresponding peak in empty SMALPs (S_1_). P_1_ exhibits a high‐field shift of 0.66 ppm and is narrower than S_1_. The second peak (P_2_) closely resembles S_2_ from the empty SMALPs, consistent with ligand associated with the lipid environment; it undergoes only a minor high‐field shift of 0.09 ppm.

A third peak (P_3_) absent in the POPC‐SMALP spectrum, appears at 50.22 ± 0.06 ppm. Compared to the analogous signal in liposomes, P_3_ is approximately tenfold broader, indicating increased structural heterogeneity, slower molecular dynamics, or both. This newly emerging, high‐field‐shifted population is most plausibly assigned to the protein‐bound ligand based on comparison with the other spectra. Its broad linewidth suggests substantial conformational flexibility of the ligand within the binding pocket—an observation that may appear counterintuitive for an antagonist at first glance. Moreover, the most downfield‐shifted peak, P_1_, differs substantially from S_1_ observed in empty SMALPs: it appears at higher field and is noticeably narrower.

This raises the question of whether P_1_, like S_1_, could originate from a SMA‐associated ligand population. However, because a smaller amount of ligand was used in the protein‐containing preparation, it is likely that such a population is simply no longer detectable since due to its higher affinity, the protein binding pocket will be occupied first. Moreover, it is improbable that the presence of the protein would shift the ligand toward the SMA polymer and thereby increase the SMA‐associated population. A titration could support the co‐existence of P_1_ and S_1_ in the NK2‐SMALP spectrum, as a strong excess of ligand should broaden the down‐field region. However, it was omitted in this study as it would be detrimental for the characterization of the other resonance due to the broad linewidth of S_1_.

Another possibility is that this peak represents a second protein‐bound population. This could originate from an additional allosteric binding site or from slow exchange between different ligand poses within the orthosteric binding pocket. Although validating either scenario would require further investigation, it is noteworthy that the only other NMR characterization of a fluorinated antagonist in this receptor family—performed on the closely related substance P receptor—reported similar dynamic behaviour for the antagonist aprepitant [15, 81].

Consequently, these observations support the notion that dynamic exchange between different binding poses is an inherent feature of GPCRs, and that the high sensitivity of ^19^F NMR offers a powerful means to detect and characterize these effects while distinguishing ligand populations residing in different environments. ^19^F ultrafast MAS is certainly a promising methodology for addressing such questions in larger molecular systems without size limitations, but the limited sample volume still poses significant sensitivity challenges that must be taken into account when designing such studies. Future progress could include the routine use of additional ^1^H‐^19^F decoupling, which was technically not possible in this work and ultrafast MAS alone should be sufficient for effective decoupling. However, one previous study showed additional small resolution gains when decoupling was used in solids [82].

## 3 Conclusion

We demonstrate the detergent‐free encapsulation of the active rat NK2 receptor into SMALPs and evaluate practical considerations relevant to SMALP preparation. Antagonist binding to NK2‐SMALPs was probed using ^19^F ultrafast MAS NMR. Our results highlight both the feasibility of this approach and the pronounced environmental sensitivity of ^19^F nuclei, which enables resolution of distinct ligand populations and provides insight into ligand plasticity within the orthosteric binding pocket of this tachykinin receptor family member. In addition, the ^19^F spectra reveal clear differences in ligand–lipid interactions between liposomes and SMALPs, underscoring the need for more systematic characterization of SMALP physicochemical properties and the experimental variables that shape them. Such studies will help further establish this versatile detergent‐free method for membrane‐protein purification and expand its applicability. More broadly, ^19^F NMR offers a powerful means of probing allosteric ligands bound to GPCRs or other challenging targets within complex membrane environments.

## Supporting Information

Additional supporting information can be found online in the Supporting Information section. The authors have cited additional references within the Supporting Information [83, 84]. **Supporting Fig. S1.** SDS‐PAGE of SEC population P in Figure 1C, which was subjected to LC‐MS analysis. **Supporting Table S1.** Particle size and polydispersity of NK2‐SMALP after different solubilisation conditions. Errors are determined from the fit which was derived from measurement triplicates from *n* = 1. **Supporting Table S2.** Affinities of endogenous ligands obtained by tryptophan fluorescence of NK2‐SMALP. Dissociation constants were obtained by fitting the measured values in Figure 5B with a one‐site binding model. The coefficient of determination *R* ^2^ of the fits is shown in the right column. Errors are obtained from measurement triplicates from *n* = 1. **Supporting Table S3.** Ligand‐dependent melting temperatures of NK2‐SMALP. Errors are obtained from measurement triplicates from *n* = 1. **Supporting Table S4.** Results of the deconvolutions of the spectra of GR159897 in POPC liposomes, POPC‐SMALP and bound to NK2‐SMALP. Errors are obtained by the fitting procedure. Results are obtained from *n* = 1.

## Conflicts of Interest

The authors declare no conflicts of interest.

## Supporting information

Supplementary Material

## Data Availability

The data that support the findings of this study are available from the corresponding author upon reasonable request.
