# Supplementary material for: Detection of an Antagonist Bound to the Neurokinin a Receptor in Styrene–Maleic Acid Lipid Particles by 19F Ultrafast Magic‐Angle Spinning Nuclear Magnetic Resonance Spectroscopy
Source: Chembiochem. 2026 Mar 23;27(6):e202500963. doi: 10.1002/cbic.202500963 (PMC13007275; doi:10.1002/cbic.202500963)
Supplement: Supplementary file 1 — Supplementary Material [file CBIC-27-e202500963-s001.pdf]

Supporting Information

Detection of an Antagonist bound to the neurokinin A receptor in SMALPs by ^{19}F ultra-fast MAS NMR Spectroscopy

Samuel Seidl[a], Aditya Prasad Patra[b, d], Chengkang Li[c, e], Johanna Becker-Baldus[a], Stefanie Kaiser[c], Christoph Reinhart[b, f], and Clemens Glaubitz*[a]

[a] S. Seidl, Dr. J. Becker-Baldus and Prof. Dr. C. Glaubitz
Institute for Biophysical Chemistry and Center for Biomolecular Magnetic Resonance (BMRZ), Goethe University Frankfurt
Max-von-Laue-Straße 9, 60438 Frankfurt am Main, Germany;
E-mail: glaubitz@em.uni-frankfurt.de

[b] Dr. A. P. Patra, Dr. C. Reinhart
Max Planck Institute of Biophysics
Max-von-Laue-Straße 3, 60438 Frankfurt am Main, Germany

[c] Dr. C. Li, Prof. Dr. S. Kaiser
Institute for Pharmaceutical Chemistry, Goethe University Frankfurt
Max-von-Laue-Straße 9, 60438 Frankfurt am Main, Germany

[d] current address: UMass Chan Medical School, University of Massachussetts, 55 N Lake Ave, Worcester, MA, USA, 01655
[e] current address: Pediatric Cancer Metabolism Laboratory and Division of Division of Oncology, Children's Hospital Zurich and Children's Research Center, University of Zurich August-Forel-Strasse 51, 8008 Zurich, Switzerland

[f] current address: BioCopy AG, Hochbergstrasse 60F, 4057 Basel, Switzerland

WILEY-VCH

Methods

Preparation of SMA

SMA was prepared as described by Kopf *et al.* with slight deviations [1]. Briefly, SMA anhydride (2:1) (abcr Chemie) were autoclaved in a 10% suspension in 1 M NaOH for three times. The hydrolysed SMA were then precipitated by the addition of concentrated HCl until a pH under five was achieved. Thereafter, the SMA were centrifuged at 10,500 × g for 20 min or 7,500 × g for 30 min. The supernatant was discarded and the SMA pellet was resuspended in 10 mM HCl for washing. This step was repeated three times. After the final wash, the pellet was lyophilised. The lyophilised powder was dissolved in SMA buffer (20 mM Tris pH 8, 150 mM NaCl) in order to yield a 10% stock solution.

Expression and purification of rat NK2

A stable *P. pastoris* cell line was used as described in an earlier study [2]. The receptor (Uniprot: P16610) carries an N-terminal His$_{10}$-tag followed by a FLAG tag and a TEV protease cleavage site, as well as a C-terminal biotinylation domain. For expression, cells were plated onto YPD-Agar plates from a glycerol stock and incubated at 30 °C for two days. Then a colony was used to inoculate 20 ml YPD medium. The pre-culture was incubated at 30 °C and 130 rpm overnight. After one day, the culture was transferred to 1 l BMGY medium and further incubated at 30 °C overnight. The BMGY culture was grown until it reached a target OD$_{600}$ of 5-7. For induction, the BMGY culture was centrifuged at 1000 × g for 10 min and resuspended in BMMY medium to reach an OD$_{600}$ of one. The culture was then incubated at 20 °C at 90 rpm for 48 h with addition of 0.5% MeOH every 12 h. The cells were then harvested by centrifugation at 5000 × g for 10 min and resuspended in 10 ml breaking buffer (Potassium phosphate buffer pH 7.5, 500 mM NaCl, 10% glycerol, 1 mM EDTA and 1 mM PMSF) per gram membrane. The cells were lysed by three passages through a high-pressure homogenizer (Stansted) at 1.5 kBar. The lysate was cleared by centrifugation at 5000 × g for 10 min. The membranes were then prepared by ultracentrifugation at 200,000 × g for

1:30 h. For solubilisation, the membrane preparation was resuspended in NK2 suspension buffer (20 mM Tris, 500 mM NaCl, 1 mM DTT and 1 mM PMSF, 1 unit of Kex-2 (Peprotech), 2% SMA) and solubilised for 2 h at room temperature. Then, the insolubilized membranes were removed by ultracentrifugation at 250,000 × g for 1 h. The supernatant was incubated with 200 µl loose Ni-NTA resin per gram membrane at 4 °C overnight. The next day, the bound SMALPs were washed with 30 CV purification buffer (20 mM Tris, 500 mM NaCl, 10% glycerol, 1 mM TCEP) supplemented with 10 mM imidazole. Then, the protein was eluted with purification buffer with 300 mM imidazole. 500 µg of the eluate was either subjected to size-exclusion chromatography (Superdex 200 Increase 10/300) for analysis or the imidazole was removed by a PD-10 desalting column (Cytiva) when the protein was prepared for downstream assays or NMR measurements.

DLS and nanoDSF measurements

DLS and nanoDSF measurements were measured using a Prometheus Panta (NanoTemper Technologies, Munich, Germany). For nanoDSF, a temperature gradient of 1 °C/min from 30 °C to 80 °C was applied. Intrinsic tryptophan fluorescence was monitored at 330 nm and 350 nm. Data analysis was performed using the manufacturer's software, and thermal transitions were identified by plotting the first derivative of the 350 nm fluorescence intensity curves. 8 µM of NK2 was incubated with 10 µM ligand for 2 h at room temperature before the measurements were performed.

LC-MS analysis

After SEC purification of the protein, the Urea was dissolved in the sample to reach a concentration of 8 M. The sample was incubated for 30 min at room temperature. Then, the sample was diluted with 10 mM ammonium acetate pH 7 to reach a final concentration of 1 M urea. For each 5 µg of protein, 100 ng of trypsin was added. The digest was performed at 35 °C for 16 h. The digest was then stored at -20 °C until the measurement. Protein digests were then filtered through a 10kDa cut-off filter (VWR, cat # 516-0229) with centrifuge at 14,000 g for 10 min. Protein digests were reduced and alkylated with tris(2-carboxyethyl)phosphine (TCEP, final concentration 15 mM) and freshly prepared chloroacetamide (CAA, final concentration 40 mM) by incubating at dark for 30 min, respectively. Sample was then acidified with 5% formic acid before LC-MS analysis.

Proteomic analysis was performed on an Orbitrap Q Exactive plus (Thermo Fisher Scientific, Waltham, MA, USA) coupled to an UltiMate™ 3000 Nano-HPLC via Nanospray Flex ion source (Thermo Fisher Scientific, Waltham, MA, USA). Mobile phase A consisted of 0.1% formic acid and mobile phase B consisted of 0.1% formic acid in acetonitrile. Protein digests were first loaded on a PepMap™ Neo Trap Cartridge (Thermo Fisher Scientific, Waltham, MA, USA) at 20 µl min^{-1} for 5 min using 100% mobile phase A and subsequently reverse eluted onto an Acclaim™ PepMap™ 100 C18 analytical column (150 mm × 0.075 mm, 2 µm, 100 Å) at a flow rate of 0.3 µl min^{-1}. The gradient started with 5% B and was then increased linearly and reached 50% B at 40 min. B% ramped (curve = 7) up to 99% B at 42 min and remained until 50 min. B% dropped down to 0% within 1 min and lasted till the end of the gradient for re-equilibration. The column oven was set to at 30°C. The mass spectrometer was operated in full-MS/data-dependent MS^2 (dd-MS^2) mode at positive ion mode. 2.5 kV Spray voltage, 275 °C capillary temperature, 10 Arb Aus Gas, and 50 S-lens RF levels were selected for the ion source. Resolution for both MS1 and MS2 was set at 35,000, isolation window was 1.7 m/z, the detail parameters were as follows. At MS1: AGC target: 1e6, maximum IT: 25 ms, scan range 250 to 1800 m/z. At MS2: AGC target: 2e4, maximum IT: 50 ms, scan range 200 to 2000 m/z, TopN: 15, NCE: 28, dynamic exclusion: 10s.

Proteomic data evaluation

Proteomic raw data from the LC-MS measurement was analysed using MaxQuant (version 1.6.5.0) using the following sequence:

DYKDDDDKDHHHHHHHHHHGDRENLYFQGGSMGTRAIVSDANILSGLESNATGVTAFSMPGWQLALWATAYLA
LVLVAVTGNATVIWIILAHERMRTVTNYFIINLALADLCMAAFNATFNFIYASHNIWYFGRAFCYFQNLFPITAMFVSI
YSMTAIAADRYMAIVHPFQPRLSAPSTKAIIAGIWLVALALASPQCFYSTITVDEGATKCVVAWPNDNGGKMLLLY
HLVVFVLIYFLPLLVMFGAYSVIGLTLWKRAVPRHQAHGANLRHLQAKKKFVKAMVLVVLTFAICWLPYHLYFILGT
FQEDIYYHKFIQQVYLALFWLAMSSTMYNPIIYCCLNHRFRSGFRLAFRCCPWVTPTEEDRLELTHTPSLSRRVNR
CHTKETLFMTGDMTHSEATNGQVGSPQDGEPAGPICKAQATSEFENLYFQGQFGGGTGGAPAPAAGGAGAGK
AGEGEIPAPLAGTVSKILVKEGDTVKAGQTVLVLEAMKMETEINAPTDGKVEKVLVKERDAVQGGQGLIKIG

WILEY-VCH

Database search result was validated in Skyline software (ver. 25.1.0.237, University of Washington, Seattle, WA, USA) by verifying retention time, isotopic correlation, and b-, y-ion distribution in the MS^2 spectra. The proteomic data are available *via* the ProteomeXchange Consortium (www.ebi.ac.uk, login with accession number PXD071082 and access code DYZk5hTALpqk).

Fluorescence spectroscopy

The tryptophan fluorescence spectrum was recorded on a JASCO FP-6500. 8 µM of NK2-SMALP was excited at 280 nm and emission was recorded from 300 nm to 400 nm. For the ligand-bound spectrum, 10 µM NKA were added and incubated for 2 h at room temperature. Ligand-dependent tryptophan fluorescence measurements were carried out in a plate reader format on a ClarioStar plus (BMG Labtech). NK2-SMALP at a final concentration of 200 nM and a final volume of 200 µl was mixed with increasing concentrations of peptide ligand and incubated at room temperature for two hours. For each sample a spectrum ranging from 325 nm to 450 nm were recorded. Measurements were performed in triplicates. For K_D determination, the intensity at 325 nm was plotted against the ligand concentration and fitted with a one-site binding model (OriginPro).

NMR sample preparation

In order to form POPC-SMALP, POPC (Avanti Lipids) was dissolved in a chloroform/methanol (9:1 vol/vol) mixture and dried under nitrogen followed by overnight rotary evaporation to completely remove the solvent. The dried lipids were resuspended in SMA buffer at a concentration of 4 mg/ml. Then, a 10% SMA stock solution was titrated until the suspension was clear. Then, 300 µM GR159897 from a 10 mM stock (in DMSO) was added to the formed SMALP effectively reaching a ligand-to-lipid ratio of 1:15 (mol/mol) and incubated for 2 h at room temperature. The formed SMALP can be separated from free SMA and free ligand by sedimenting the solution into the MAS rotor using a home-built tool at 30,000 rpm in a SW50.1 rotor for 16 h.

For NK2-SMALP, a 230 µL solution of 75 µM NK2 in SMALPs was incubated with 80 µM GR159897 for 2 h at room temperature before the sample was centrifuged into the MAS rotor as described above. Subsequently, the MAS rotor contained then an estimated amount of max. 0.5 mg NK2.

In order to characterise GR159897 in POPC liposomes, the lipid suspension was incubated with 300 µM GR159897 for 2 h. Then, the liposomes concentrated by centrifuging at 16,200 × g for 10 min. The lower aqueous phase was removed and the lipid suspension was injected into the MAS rotor.

NMR spectroscopy

All experiments were performed on a Bruker Avance III wide-bore ssNMR spectrometer operating at 850 MHz ^{1}H Larmor frequency (800 MHz ^{19}F Larmor frequency) equipped with a Bruker 0.7 mm HCN probehead tunable to ^{19}F on the ^{1}H channel. If not indicated otherwise, experiments were performed at 100 kHz MAS. In order to ensure a similar sample temperature for varying MAS frequencies, the proton chemical shift of water was monitored. Spectra were referenced to 0.5 mM buffered TFA which was added before sedimentation into the MAS rotor.

^{19}F spectra were acquired using a Hahn echo pulse sequence with an inter pulse delay of 100 µs in order to reduce background signals from the probehead. 90° pulse duration was 1.05 µs. Between 32,000 and 51,000 transients were acquired with an acquisition time of 50 ms. For the ligand in liposomes and in presence of NK2 a recycle delay of 3.0 s was used and for the ligand in POPC-SMALP 1.5 s. During processing, the FID was typically cut after 10 ms. For the samples containing only the ligand, 50 Hz exponential linebroadening was applied. For the sample with NK2 and POPC-SMALP, 300 Hz exponential linebroadening was applied. For the liposome sample, 100 Hz exponential linebroadening was applied.

To deconvolute the processed 1D spectra, all peaks were modelled using a Lorentzian line shape using OriginPro. We first performed a two-state deconvolution on the sample containing only the ligand in SMALP. The parameters of the lipid-associated obtained from this fit were then used as starting points to model the third peak observed in the spectrum of the ligand–receptor sample. The parameters for the SMA-associated peak were not suitable to model the ligand-receptor spectrum, as they lead to strong deviations in the lipid-associated peak.

WILEY-VCH

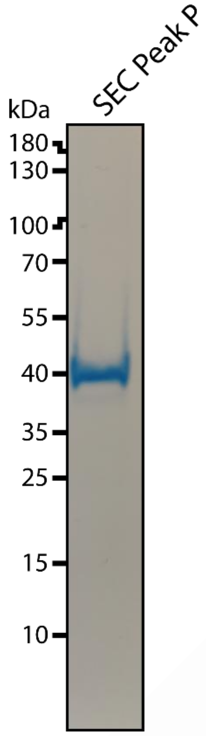

Figure S1. SDS-PAGE of SEC population P in Figure 1C which was subjected to LC-MS analysis.

Table S1. Particle size and polydispersity of NK2-SMALP after different solubilisation conditions Table caption. Errors are determined from the fit which was derived from measurement triplicates from n = 1.

[SMA] [%], [Membrane] [mg/ml]	Radius [nm]	Polydispersity index
2, 50	29.32 ± 1.19	0.18 ± 0.07
2, 100	25.15 ± 0.77	0.35 ± 0.06
4, 100	29.67 ± 0.79	0.18 ± 0.03

Table S2. Affinities of endogenous ligands obtained by tryptophan fluorescence of NK2-SMALP. Dissociation constants were obtained by fitting the measured values in Figure 5B with a one-site binding model. The coefficient of determination R^2 of the fits is shown in the right column. Errors are obtained from measurement triplicates from n = 1.

WILEY-VCH

Ligand	K_D [nM]	R^2
Neurokinin A	6.65 ± 2.00	0.96
Neurokinin B	6.94 ± 2.18	0.95
Substance P	142.13 ± 70.77	0.93

Table S3. Ligand-dependent melting temperatures of NK2-SMALP.

Errors are obtained from measurement triplicates from n = 1.

Ligand	T_M [°C]
Apo	52.95 ± 0.06
Neurokinin A	53.16 ± 0.19
Neurokinin B	53.13 ± 0.11
Substance P	53.49 ± 0.05
GR64349	53.15 ± 0.3
GR159897	53.72 ± 0.21
Saredutant	53.44 ± 0.16

Table S4. Results of the deconvolutions of the spectra of GR159897 in POPC liposomes, POPC-SMALP and bound to NK2-SMALP. Errors are obtained by the fitting procedure. Results are obtained from n = 1.

WILEY ◆ VCH

GR159897…	Peak	^{19}F chemical shift [ppm]	Linewidth [Hz]	Integral [a.u.]	R^2
in liposomes	1	-50.29 ± 0.00	176 ± 2	0.33 ± 0.00	0.95
in SMALP	S_1	-47.12 ± 0.06	2464 ± 120	1.14 ± 0.07	0.96
in SMALP	S_2	-49.02 ± 0.01	1112 ± 19	1.87 ± 0.04	0.96
bound to NK2	P_1	-47.82 ± 0.05	1720 ± 72	0.88 ± 0.07	0.99
bound to NK2	P_2	-49.08 ± 0.02	1088 ± 80	1.36 ± 0.10	0.99
bound to NK2	P_3	-50.04 ± 0.07	2424 ± 48	2.46 ± 0.17	0.99

References

[1] A. H. Kopf, M. C. Koorengevel, C. A. van Walree, T. R. Dafforn and J. A. Killian, *Chem Phys Lipids* **2019**, *218*, 85-90.

[2] N. Andre, N. Cherouati, C. Prual, T. Steffan, G. Zeder-Lutz, T. Magnin, F. Pattus, H. Michel, R. Wagner and C. Reinhart, *Protein Sci* **2006**, *15*, 1115-1126.

WILEY-VCH
